# MEDICAL AID IN DYING: DOES POLICY PREVENT SUFFERING AT THE END OF LIFE?

**DOI:** 10.1093/geroni/igac059.2510

**Published:** 2022-12-20

**Authors:** Todd Becker, Nancy Kusmaul, Allison Gibson, Cara L Wallace

**Affiliations:** University of Maryland Baltimore County, Baltimore, Maryland, United States; University of Maryland Baltimore County, Baltimore, Maryland, United States; University of Kentucky, Lexington, Kentucky, United States; Saint Louis University, Saint Louis, Missouri, United States

## Abstract

Medical aid in dying (MAID) reflects the legal provision for qualifying, terminally ill individuals to receive a prescription from their medical provider for self-ingestion to hasten death. This policy analysis examines current MAID policy with a focus on MAID’s intended relief of suffering. Using Wallace’s (2015) policy model, we evaluated MAID on: policy description, historical context, development of the policy, efficiency, alternative programs, effectiveness, and unintended consequences. Policy description: Currently legal in 10 jurisdictions (nine states and Washington, DC), common goals include relieving suffering and promoting self-determination. Historical context: Following a failed attempt at federal legalization, the U.S. adopted an incremental, jurisdiction-based approach to legalization beginning in Oregon (1994). Development of the policy: Although all statutes imitate Oregon’s model, subtle differences exist. Efficiency: Although each policy includes waiting periods that vary in sequential order and duration, revisions have targeted waiting periods to improve efficiency. Alternative programs: Frequently considered an alternative to MAID, existing statutes encourage providers to discuss palliative options. Effectiveness: Despite appearing effective in promoting self-determination, MAID-related complications (e.g., vomiting) may exacerbate suffering. Unintended consequences: MAID’s requirement of clinician participation may place providers in situations that challenge their self-determination and provoke distress. In sum, many questions remain unanswered regarding MAID’s effectiveness and efficiency. Thus, more data are needed. Ethical implications will continue to be debated, though policy implications (e.g., who uses MAID, the number of unique providers, the emergence of complications, those present during death) greatly impact implementation and overall choices to limit suffering at the end of life.

